# Ionizing Radiation Selectively Reduces Skin Regulatory T Cells and Alters Immune Function

**DOI:** 10.1371/journal.pone.0100800

**Published:** 2014-06-24

**Authors:** Yu Zhou, Houping Ni, Klara Balint, Jenine K. Sanzari, Tzvete Dentchev, Eric S. Diffenderfer, Jolaine M. Wilson, Keith A. Cengel, Drew Weissman

**Affiliations:** 1 Division of Infectious Diseases, Department of Medicine, University of Pennsylvania, Philadelphia, Pennsylvania, United States of America; 2 Department of Radiation Oncology, University of Pennsylvania, Philadelphia, Pennsylvania, United States of America; 3 Department of Dermatology, University of Pennsylvania, Philadelphia, Pennsylvania, United States of America; Louisiana State University and A & M College, United States of America

## Abstract

The skin serves multiple functions that are critical for life. The protection from pathogens is achieved by a complicated interaction between aggressive effectors and controlling functions that limit damage. Inhomogeneous radiation with limited penetration is used in certain types of therapeutics and is experienced with exposure to solar particle events outside the protection of the Earth’s magnetic field. This study explores the effect of ionizing radiation on skin immune function. We demonstrate that radiation, both homogeneous and inhomogeneous, induces inflammation with resultant specific loss of regulatory T cells from the skin. This results in a hyper-responsive state with increased delayed type hypersensitivity in vivo and CD4^+^ T cell proliferation in vitro. The effects of inhomogeneous radiation to the skin of astronauts or as part of a therapeutic approach could result in an unexpected enhancement in skin immune function. The effects of this need to be considered in the design of radiation therapy protocols and in the development of countermeasures for extended space travel.

## Introduction

Radiation therapy of the skin is used in the treatment of cutaneous T cell lymphomas (reviewed in [1]) and multiple types of skin cancer (reviewed in [2]). Typically, the energy and type of radiation results in major doses and subsequent effects in the skin. In interventional radiology procedures, large skin doses can lead to major adverse effects in skin, as well as, tissues beneath the skin [3]. In addition, nearly all radiation treatments must pass through the skin to reach their targets. Another source of radiation that primarily targets the skin are solar particle events (SPEs). Future space missions will involve extended mission durations (e.g., approximately 3 years for a voyage to Mars). In addition to the risks associated with extended missions in space, SPE and cosmic radiation that are mitigated by the Earth’s magnetic field, become important hazards to astronaut health and safety and mission success. SPEs occur when protons emitted by the Sun become accelerated to high energies during a solar flare or coronal mass ejection. SPEs result in radiation largely comprised of protons of 50 MeV energy or less, with a minor contribution of electrons, alpha particles, and heavier particles. This proton energy profile will produce an inhomogeneous dose distribution with limited penetration. It is calculated that an astronaut could receive up to 32 Gy of radiation to the skin and 2 Gy to the bone marrow during a strong SPE, if exposure occurs during extravehicular activity (EVA) [4], [5].

The skin functions to protect the body against pathogens, control evaporation, insulate, regulate temperature, synthesize vitamin D, protect against degradation of folate, and provide a waterproof surface for the body (reviewed in [6]). The skin immune system contains many different types of cells including dendritic cells, CD4^+^ and CD8^+^ T cells, γδ T cells, B cells, natural killer T (NKT) cells, macrophages, mast cells, and Langerhans cells (reviewed in [7]–[9]). While much of the early work on the immune system of the skin identified mechanisms and factors that activate immune responses, more recent studies have identified systems that control and limit inflammation and immune responses (reviewed in [7], [10]). Such systems are critical, as damage caused by uncontrolled inflammation impairs skin function. The effect of radiation on skin immune function has been extensively described for ultraviolet (UV) wavelengths (reviewed in [11]), but studies of more deeply penetrating ionizing radiation used therapeutically for malignancies are lacking.

The original genesis of this study was to determine the effect of SPE-like radiation on immune function to determine whether an astronaut’s health and mission could be put into jeopardy and to determine the mechanisms, allowing countermeasures to be developed. We identified an unexpected increase in skin immune responses mediated by a selective loss of regulatory T cells. This loss was associated with a local inflammatory state. Transient systemic decreases in T cell function due to a relative increase in regulatory T cells likely due to trafficking from the skin was also observed.

## Materials and Methods

### Ethics Statement

This study was carried out in strict accordance with the recommendations in the Guide for the Care and Use of Laboratory Animals of the National Institutes of Health. All animals and activities involved in this study were conducted under a protocol approved by the University of Pennsylvania. The Federal Government Animal Welfare Assurance number for the University of Pennsylvania is A3079-01. Facilities housing the animals involved are accredited by the Association for Assessment and Accreditation of Laboratory Animal Care, International (AAALAC) and inspected regularly by the U.S. Department of Agriculture (USDA). All efforts were made to minimize suffering.

### Irradiation of Pigs

Three 3–4 month old (9–10 kg) Yucatan mini-pigs (Sinclair BioResources, Columbia, MO) per group were individually placed in a clear cast, aerated, acrylic (Plexiglas) irradiation chamber (custom-made at the University of Pennsylvania Machine Shop) and exposed to 5, 7.5, 10, 12.5, 15 or 25 Gray (Gy) total skin dose of 6 MeV electrons delivered by a linear accelerator (Clinac iX linear accelerator, Varian Medical Systems). Sham irradiated pigs were placed in the same housing for the same amount of time and served as controls. Animals were not anesthetized for the irradiation procedure and were able to express normal postural movements throughout the procedure. The entire dose was delivered over approximately three hours, resulting in a dose rate of 1.67, 2.5, 3.3, 5, 6.7, or 8.3 Gy/hr respectively. The radiation dose was delivered in 2.5–6.25 Gy increments to one side of the long axis of the pig’s body, after which the entire irradiation chamber was rotated 180 degrees and an identical dose was delivered to the opposite side of the pig. This process was repeated until the total dose was delivered equally to both lateral sides of the pigs. The radiation dosimetry details are described elsewhere [12]. Dose depth calculations were made using a Geant4-based Monte Carlo simulation [13] of 6 MeV electrons in water.

### Irradiation of Mice

Male and female outbred ICR mice, 5–6 weeks of age, were obtained from Harlan Laboratories. For irradiation, the mice were placed in aerated plastic chambers (AMAC #530C) with dimensions of 7.30 cm×4.13 cm×4.13 cm. The chambers allowed the mice to easily turn around (reverse nose to tail direction). Gamma radiation (1 or 2 Gy) was delivered using a ^6^°Co source (Eldorado Model ‘G’ machine, Atomic Energy of Canada Ltd, Commercial Products Division). The gamma-radiation exposures were delivered in a single fraction at 50 cGy/min. Mice were exposed to total-body irradiation with 2 Gy of protons (delivered as a uniform spread out Bragg peak (SOBP)). The proton beam was produced by an IBA cyclotron system. Dosimetry verification was performed before the proton irradiations with a 2D ion chamber array (I’m*RT* MatriXX, IBA dosimetry) placed at a water equivalent depth of 13.3 cm. The proton radiation exposures were delivered in a single fraction at a dose rate of 50 cGy/min. Mouse proton irradiations have been described previously [14]–[17]. Non-irradiated control mice were placed in the same chambers for the same amount of time.

### Intradermal Challenge

Prior to and 7, 14, and 30 days post irradiation, pigs were injected intradermally on lateral non-overlapping sites with 50 µl of PBS, PHA (20 µg) in PBS, and LPS (200 µg) in PBS. Forty-eight hrs later, erythema, induration, and ulceration were measured as the average diameter. Responses were consistently symmetric. Mice were challenged with PBS or PHA (1 µg) in a volume of 25 µl by the intradermal route on shaved flank skin at the indicated times. Skin thickness (epidermis to hypodermis) of euthanized mice was measured 48 hr later using an Electronic Digital Micrometer (Marathon Management Co., Fisher scientific). Classical delayed type hypersensitivity (DTH) in mice was measured by priming with a subcutaneous injection of 300 µg ovalbumin (OVA) (Grade VI; Sigma-Aldrich) in PBS emulsified in complete Freud‘s adjuvant (1∶1) (CFA; Sigma-Aldrich), 200 µl total volume. Mice were irradiated 21 days later, and were then challenged on the indicated days post irradiation by injection of 50 µg OVA (Grade VI) in 10 µl PBS into the right ear and PBS only into the left ear. Ear swelling was measured before challenge and 48 hrs later. DTH response was calculated as described previously [18].

### Tissue Lymphocyte Isolation

Murine skin was minced into small pieces (2 mm^3^) and digested in 500 µg/ml Liberase DH Research Grade (Roche) for 30 min at 37°C. One hundred mM EDTA was added and tissue was processed into a single-cell suspension by repeated pipetting. Cells were then passed through a 70 µm cell strainer (Fisher). Total skin cells were analyzed. Spleens were processed into a single cell suspension by passage through a 70 µm cell strainer. CD25^+^ T cells were removed, where indicated, using magnetic beads, as described by the manufacturer (Invitrogen). Bead based depletion was 90–95% effective, as measured by FoxP3 staining.

### CFSE Proliferation

Murine spleen and skin lymphocytes with or without CD25 depletion were loaded with the fluorescent cytoplasmic dye carboxyfluorescein diacetate, succinimidyl ester (CFSE), as described (Invitrogen), and stimulated with plate bound anti-CD3 mAb (clone 145-2C11, BD Pharmingen). After 3 days, cells were stained with anti-CD4-APC mAb (BD) and analyzed on a FACSVantage. Percent of input cells that proliferated were calculated, as described [19].

### Flow Cytometry

Staining for CD4^+^ regulatory T cells used the Foxp3 Staining Set (FoxP3-PE, CD4-FITC, CD25-APC), as described by the manufacturer (eBioscience), and were analyzed on a FACSVantage.

### Skin mRNA Analyses

Total RNA was isolated from full thickness murine skin at the indicated time points from 3 to 6 animals per time point. Total RNA from each mouse was analyzed using the mouse inflammatory response PCR array (PAMM-077, SABiosciences), as directed by the manufacturer. Data were first corrected for housekeeping genes, averaged for each time point, and then expressed as change from untreated.

Quantitative reverse transcriptase real time PCR for murine CD3, CD4, FoxP3, and GAPDH was performed using validated primers (Real Time Primers). Total RNA using TRIzol reagent and poly(A) RNA by the Dynabeads mRNA Purification Kit (Invitrogen) was isolated. First-strand cDNAs were synthesized using M-MLV reverse transcriptase (Promega) with random primers. SYBR Green real-time PCR in an ABI 7300 (Applied Biosystem) was performed. mRNA expression was normalized to GAPDH using the Delta Ct method. Reactions were performed in triplicate for each mouse with 5 mice per time point or treatment group.

### Statistical Analyses

Mean, standard error of the mean (SEM), and Student’s t-test were calculated using Microsoft Excel. Analyses of skin genes used the Student’s t-test of the replicate 2∧(−Delta Ct) values for each gene in the control and treatment groups. Bonferroni correction to account for multiple analyses was used, where indicated.

### Data

Data was submitted to the National Space Biomedical Research Institute (NSBRI) and is freely available for review.

## Results

**Figure 1:**
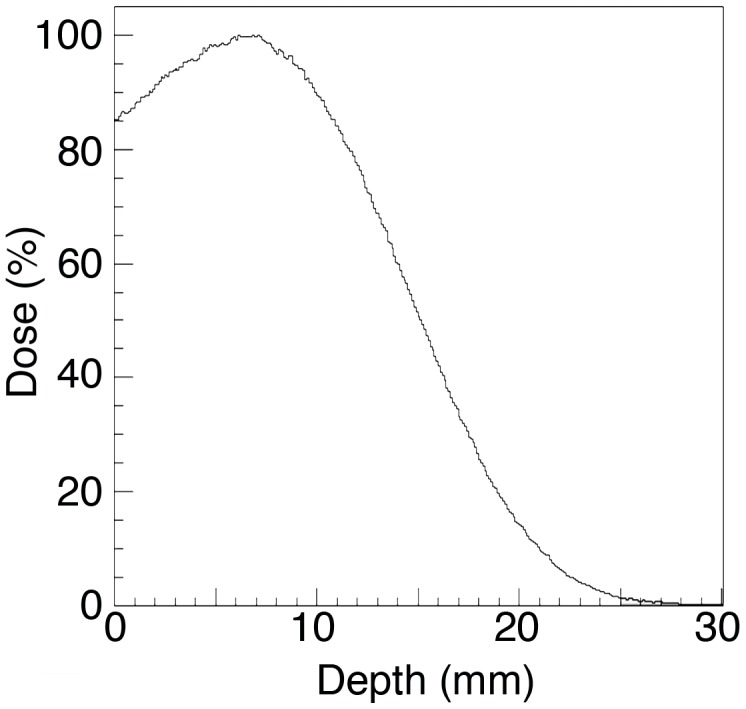
Six MeV electron radiation results in a dose to depth distribution that deposits the majority of the radiation to the skin of a pig. The percent of radiation dose versus depth using water was calculated using a Monte Carlo-based simulation algorithm (Varian Medical Systems).

We used 2 models to study the effect of radiation on skin immune function. The first used miniature pigs, as their skin histologic structure is an accurate model of human skin, but due to their cost, extensive tissue and systemic studies could not be performed. Mice were used to allow in depth investigations of alterations in immune function of the skin and draining lymphoid organs. The main difference in the delivery of radiation between the two models was the depth of penetration. Electron radiation in pigs was used as the dose distribution could be modeled to simulate the dose distribution expected from exposure to low energy, SPE-like protons [20]. The pigs were exposed to 6 MeV electrons that delivered most of the radiation up to a depth of 1.5 cm (Figure 1) similar to that of SPE-like protons. The high doses of radiation to the skin that have been estimated by Hu et al. [4] could not be delivered with gamma radiation, as the homogenous distribution of gamma-rays with deep organs receiving similar doses as the skin would result in morbidity in the pig model. The mice were exposed to gamma radiation at 50 cGy/min dose rates and to protons with a spread out bragg peak (SOBP) at a dose rate of 50 cGy/min. Gamma radiation was used to measure Relative Biological Effect (RBE) of the proton radiation and because of limited availability of the proton beam. An RBE of 1 was found based on equivalence of effect in dose response experiments. The limited depth of penetration could not be modeled in mice; thus, we used the maximal likely dose that an astronaut could receive to the bone marrow (2 Gy) during a strong SPE [4], [5].

**Figure 2:**
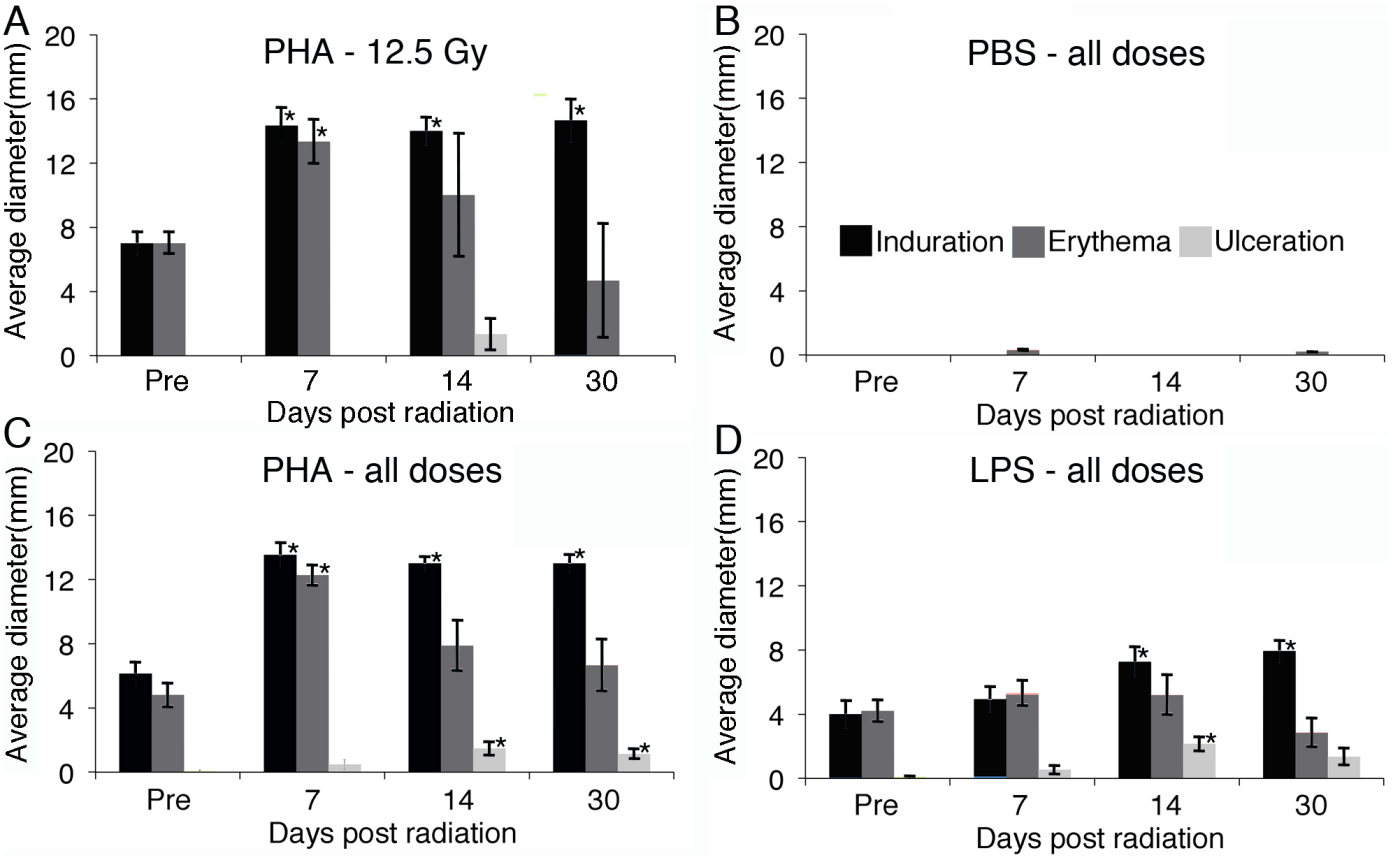
Intradermal PHA and LPS responses in miniature pigs exposed to SPE-like radiation. Local reaction to intradermal PBS, PHA, and LPS injections were measured 48(n = 3) (A) showed a statistically significant increase in the diameter of response to PHA compared to pre-irradiation. As a similar response to radiation was found at each dose, animals receiving all amounts of electron radiation (5, 7.5, 10, 12.5, 15 and 25 Gy, 3 per dose) were combined for analysis. The response to stimulation with PBS (B), PHA (C), and LPS (D) were measured as the diameter of induration, erythema, and ulceration and averages ± SEM are presented. Statistical significance compared to pretreatment values was determined by Student’s t-test, using Bonferroni’s correction for multiple comparisons, * = p<0.017.

**Figure 3:**
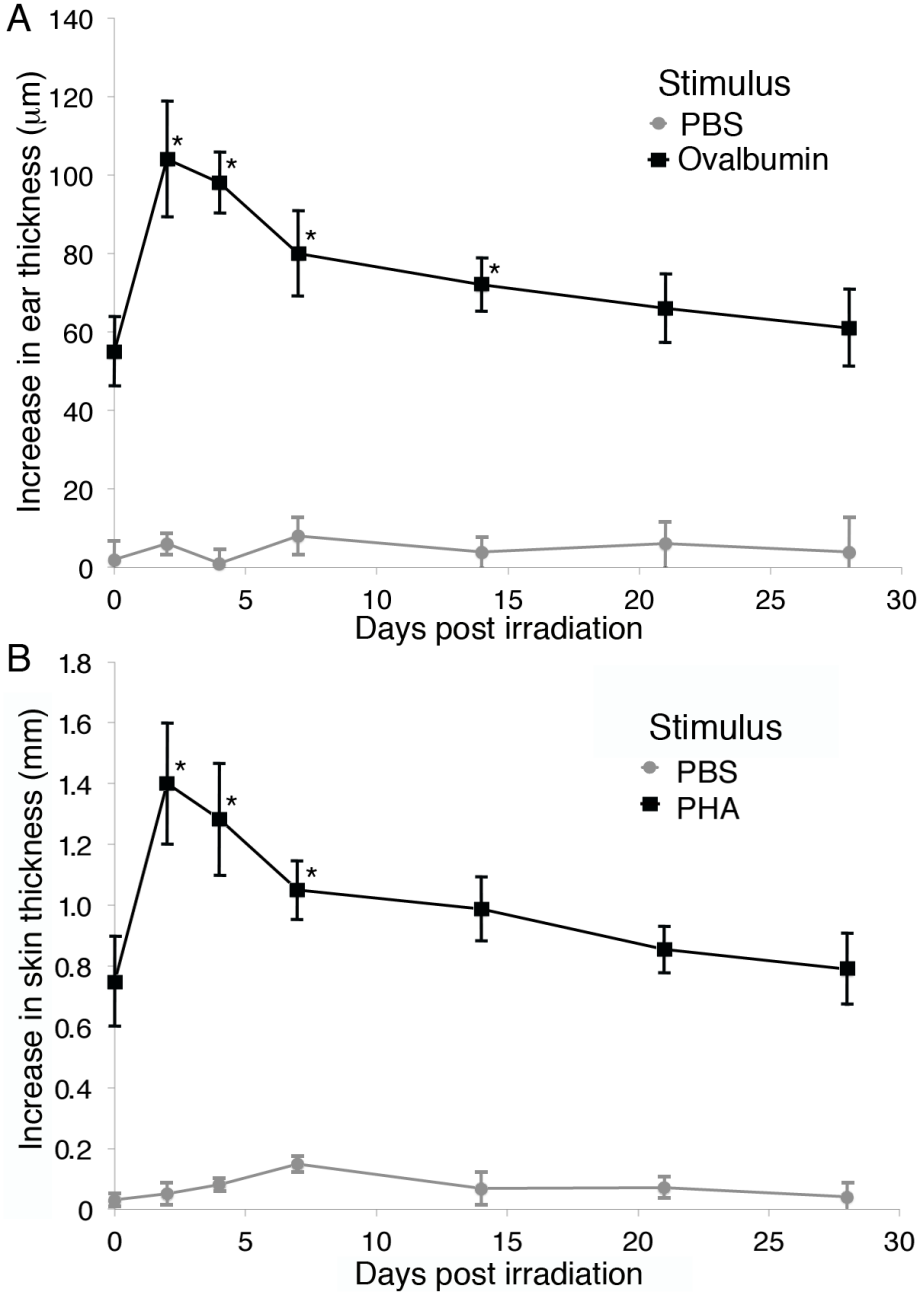
DTH and intradermal PHA responses in mice are enhanced after exposure to radiation. ICR mice were stimulated with ovalbumin in CFA, rested for 21 days, irradiated with 2-irradiation (A) or injected intradermally on the back with PHA or PBS (B). Ear and skin swelling was measured with an electronic digital micrometer and calculated, as described [18]. Five animals per time point were averaged and error bars represent the SEM. Statistical significance compared to pretreatment values was determined by Student’s t-test, * = p<0.05.

Very high doses of radiation to the skin could be expected during a strong SPE (up to 32 Gy) [4], [5]. Miniature pigs were exposed to different doses of 6 MeV electron radiation (5, 7.5, 10, 12.5, 15, and 25 Gy) and response to intradermal PHA and LPS were measured in sham irradiated animals, prior to irradiation, and at 7, 14, and 30 days post-radiation. PHA was used to mimic the response to a T cell activating antigen leading to a delayed type hypersensitivity (DTH)-like response, as has been demonstrated in multiple animal models [21]–[28]. PHA allowed us to repeatedly stimulate the same animals and obtain consistent results, where as, the use of a true DTH antigen would be complicated by subsequent memory. Responses were symmetric and recorded as the average diameter of induration, erythema, and ulceration. Figure 2A demonstrates the responses to PHA in 3 pigs that received 12.5 Gy of radiation. As similar responses were observed at all doses of radiation with no dose dependent effects, we analyzed all radiation doses together for the response to control (PBS), PHA, and LPS intradermal injections (Figure 2B–D, respectively). A significant enhancement in the response to PHA was observed at all time points after irradiation. The responses to LPS were not significantly elevated at day 7, but became significant at 14 days post irradiation. The appearance of ulceration after radiation was noted for both PHA and LPS. It is likely that ulceration occurred as part of the enhanced response post-irradiation, but we cannot exclude a direct contribution from radiation-induced damage. Although, if this were responsible for the ulceration, we would have expected to see an increase in ulceration with the amount of radiation given, which was not observed. Sequential challenges of sham-irradiated pigs demonstrated similar responses to PHA and LPS over time. Mouse ear challenge with ovalbumin and skin challenged with intradermal PHA were measured using a micrometer and a similar increase in reactivity was noted after 2 Gy of gamma or proton radiation (Figure 3A and B and Figure S1A and B). Sham-irradiated mice were sequentially injected intradermally with PHA and similar levels of skin thickness were observed over 28 days. Non-irradiated control pigs and mice were placed in the same chambers for the same amount of time as the maximum dose of radiation received.

**Figure 4:**
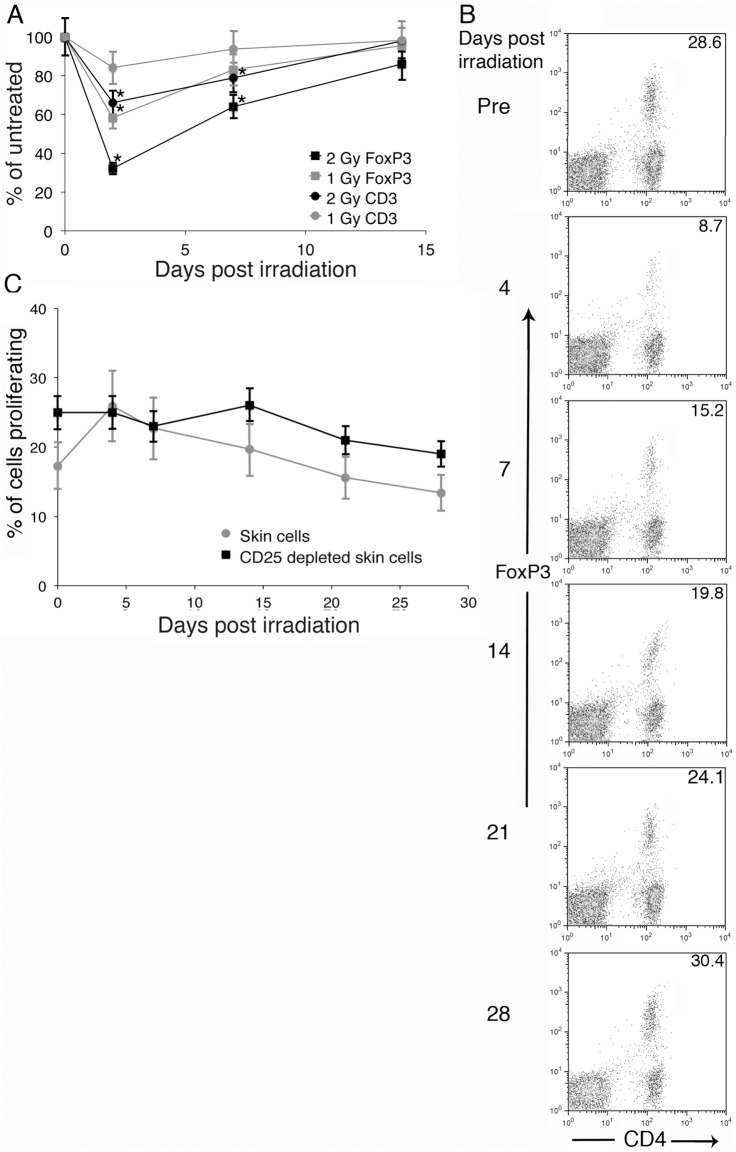
Radiation causes a loss of regulatory T cells in the skin, which leads to an increase in CD4^+^ T cell proliferation. (A) ICR mice were exposed to 0, 1, or 2 Gy of gamma radiation, skin was obtained prior to and 2, 7, or 14 days later, and poly(A) RNA was isolated. Copies of CD3 and FoxP3 mRNA were quantitated and normalized to copies of GAPDH mRNA. Data are expressed as % of unirradiated. Ten mice per group were analyzed and data are presented as mean +/− SEM. (B) Cells were obtained from the skin of pre-irradiation or gamma irradiated (2 Gy) mice at the indicated times post irradiation. Cells were analyzed for regulatory T cells (CD4^+^, CD25^+^, FoxP3^+^). (C) Skin cells were depleted with CD25 beads or not, labeled with CFSE, and stimulated with plate bound anti-CD3. The number of cells that proliferated was calculated, as described [19]. Five mice per time point were analyzed and data are presented as mean +/− SEM. Statistical significance compared to pretreatment was determined by Student’s t-test, * = p<0.05. For 4C, a significant increase in skin cell proliferation compared to pretreatment was observed at 4 and 7 days post-radiation, as noted by a *.

Comprehensive analyses of systemic immune function were not possible in the pig model system. As we observed a similar increase in the skin response to PHA and ear responses to ovalbumin in mice irradiated with 2 Gy of protons or gamma rays (Figure 3A and B and Figure S1A and B), we used the mouse model system to further understand the effects of ionizing radiation on skin immune function. A maximal dose of 2 Gy was chosen, as it is estimated to be close to the largest dose expected to reach the bone marrow during a strong SPE [4], [5] and dosing selectively to the skin in mice was not possible. To determine whether a selective loss of Tregs was responsible for the enhanced PHA and LPS responses in the miniature pigs (Figure 2) and ovalbumin and PHA responses in mice (Figure 3 and Figure S1), poly(A) purified RNA was isolated from murine skin prior to and at 2, 7, and 14 days post-irradiation. Quantitative PCR measurement of CD3, CD4, FoxP3, and GAPDH mRNA was performed and values were normalized to GAPDH. A significant reduction in FoxP3 mRNA, the master regulator in the development and function of regulatory T cells, in a dose dependent manner was observed (Figure 4A). Smaller decreases in CD3 and CD4 (data not shown) mRNA were observed, demonstrating that FoxP3 positive cells were being selectively lost. Sham-irradiated controls demonstrated similar levels of CD3, CD4, and FoxP3 mRNA relative to GAPDH over the duration of the experiment.

To confirm the effect of the loss of Treg cells from the skin on immune function, murine skin was obtained before and at 4, 7, 14, 21, and 28 days post-irradiation. Single cell suspensions were obtained by enzymatic digestion. The cells were analyzed for CD4, CD25, and FoxP3 by flow cytometry. A significant decrease in the percent of CD4^+^ T cells expressing FoxP3 was observed through 14 days post-irradiation. The greatest loss was observed at 4 days post-irradiation, with a slow increase in Tregs over the next 24 days (Figure 4B). All FoxP3 positive cells were CD25 positive (data not shown). A suboptimal amount of anti-mouse CD3 that stimulates 25–50% of maximum was coated onto plates and CFSE-labeled skin lymphocytes were added. The number of proliferating CD4^+^ cells was calculated as described [19] and expressed as the percent of added CD4^+^ T cells that proliferated. The proliferation of skin CD4^+^ T cells increased with the radiation-induced loss of Tregs (Figure 4C), demonstrating a functional effect. Depletion of CD25^+^ cells with magnetic beads (90–95% removal of FoxP3^+^ cells) prior to CD3 stimulation resulted in similar levels of proliferation at all times before and after irradiation (Figure 4C) demonstrating Tregs were responsible for the altered T cell function resulting from radiation. Sham-irradiated mice gave similar levels of CD4^+^, CD25^+^, FoxP3^+^ cells and similar levels of proliferation at all time points.

**Figure 5:**
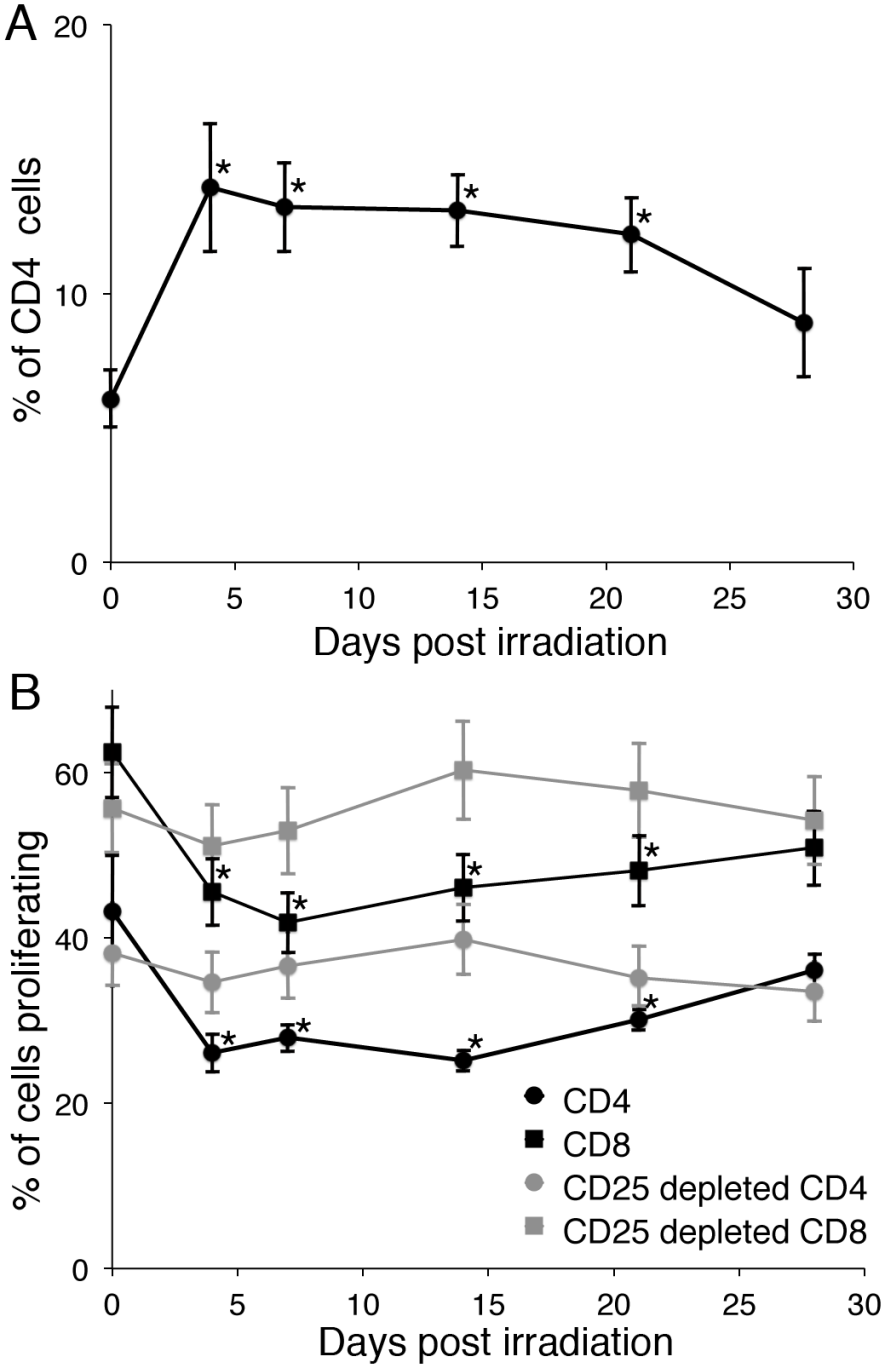
Radiation induces an increase in splenic regulatory T cells with a decrease in T cell proliferation. Mononuclear cells were obtained from the spleen of sham or gamma irradiated (2 Gy) mice at the indicated times pre and post irradiation. Cells were analyzed for regulatory T cells (CD4^+^, CD25^+^, FoxP3^+^) and expressed as percent of total CD4^+^ T cells (A) or CD25 bead depleted or not and labeled with CFSE and stimulated with plate bound anti-CD3 (B). The number of cells that proliferated was calculated, as described [19]. Five mice per time point were analyzed and data are presented as mean +/− SEM. Statistical significance compared to pretreatment was determined by Student’s t-test, * = p<0.05.

The selective loss of skin Tregs could be due to radiation induced apoptosis or alterations in trafficking. We examined splenic lymphoid cells and in the setting of a decrease in the total number of cells and T cells (40%+/−15%), we found that with the loss of skin Tregs in irradiated mice, there was a significant increase in the percent of Tregs in the spleen (Figure 5A). This suggested the possibility that Tregs were trafficking out of the skin to lymphoid organs, as it would be unexpected but not impossible that skin Tregs have an increased sensitivity to radiation while splenic Tregs had a decreased sensitivity, as the radiation delivered to the mice resulted in a homogeneous distribution with equal amounts delivered to skin and internal organs. The increase in Tregs in the spleen led to a drop in the proliferation of anti-CD3-activated CD4^+^ and CD8^+^ T cells (Figure 5B), demonstrating that the increase in splenic Tregs had a functional effect. The depletion of Tregs from splenic cells resulted in similar levels of proliferation at all times demonstrating that Tregs were responsible (Figure 5B). Sham-irradiated mice examined over 28 days post enclosure residence demonstrated similar levels of CD3^+^, CD25^+^, FoxP3^+^ cells and similar levels of CD4^+^ and CD8^+^ T cell proliferation at each time point.

**Figure 6:**
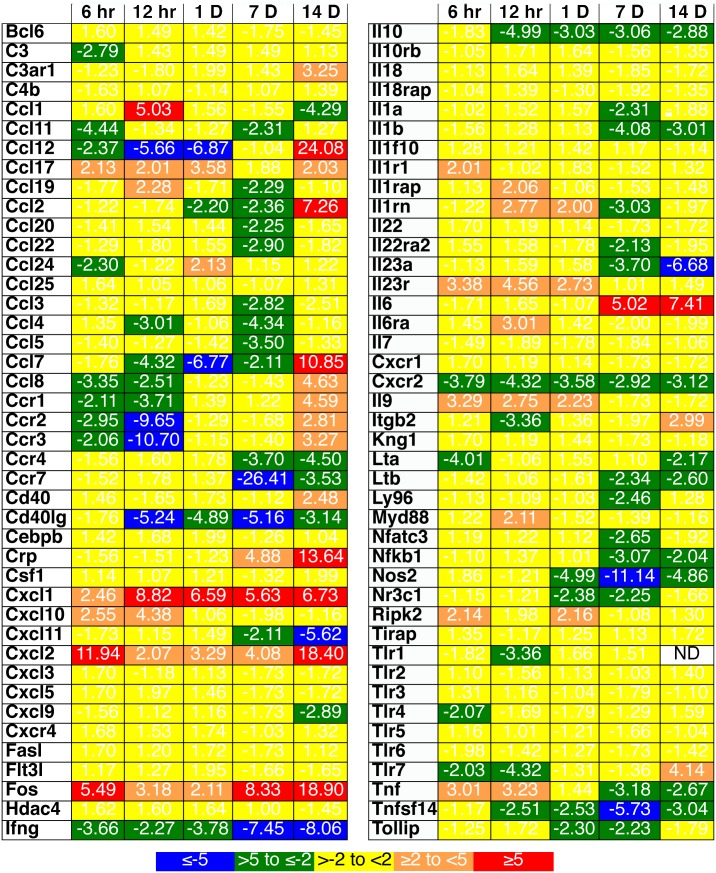
A long-lived inflammatory state is induced in murine skin after 2 Gy of gamma radiation. Total RNA from murine skin prior to and at the indicated time points after gamma radiation (2 Gy) were obtained. Quantitative reverse transcriptase PCR arrays were performed. Three to 6 mice per time point were analyzed. All samples demonstrated no DNA contamination or inhibitors of PCR and were first normalized for housekeeping genes, averaged for each time, and expressed as relative change versus untreated. P-values were calculated using the Student’s t-test of the replicate 2∧(−Delta Ct) values for each gene in the control versus treatment groups. The calculation of p-values for each gene in general found significance below 0.001 for changes greater than 2-fold.

As it has been demonstrated that skin Tregs increase their trafficking to draining lymphoid organs after local inflammation [29], quantitative PCR analyses for murine inflammatory genes were performed on control and irradiated skin (2 Gy gamma radiation) at 6, 12, and 24 hrs and 7 and 14 days post-irradiation. An initial acute inflammatory profile characterized by increases in the expression of genes associated with inflammatory cytokines (TNF-α) and chemokines (CCL1, CCL17, CXCL1, and CXCL2) was observed (Figure 6). This was followed by long lasting chronic inflammation, with increases in the expression of IL-6, C-reactive protein (crp), and immunoregulatory chemokine genes. The expression of genes normally associated with an inflammatory state in T cells; IFN-γ, CD40 ligand, CCR7, lymphotoxin (LT)A, and LTB and with regulatory T cell activity, IL-10, was reduced through day 14. A reduction in the gene expression patterns for chemokines that recruit lymphocytes in the setting of local lymphopenia was noted until day 14, when these chemokine genes were upregulated, and a decrease in gene expression for the skin homing chemokine receptor, CCR4, used by regulatory T cells, was observed through day 14.

## Discussion

Using a model of SPE radiation, we observed alterations affecting the skin immune system that leads to systemic immune changes. After 2 Gy of gamma radiation to mice, there was an acute decrease of all lymphocytes from the skin and spleen and a selective loss of regulatory CD4^+^ T cells from the skin with an increased frequency of Tregs in the spleen. In the mouse, the skin CD4^+^ T cells had an increased response to mitogenic stimulation due to the decrease in regulatory T cells, while the opposite occurred in the spleen. These in vitro findings paralleled the in vivo PHA and LPS intradermal responses observed in pigs that received 6 MeV electron radiation and the response to ovalbumin and PHA in gamma-irradiated mice. Irradiation resulted in a chronic inflammatory state in the skin that was likely responsible for the selective loss of regulatory T cells, as has been observed after inflammation induced by 2,4-dinitro-1-fluorobenzene (DNFB) stimulation [29]. While there was a decrease in all lymphocytes after irradiation, the enhanced selective loss of regulatory T cells from skin, with an increase in the relative frequency of regulatory T cells in the spleen, suggested that increased Treg trafficking occurred. Immunologically, hyper-responsiveness was found in the skin of both mice and pigs, as measured both in vivo and in vitro.

It has been previously observed in studies of T cell trafficking out of the skin in mice that, at steady state, approximately half are regulatory T cells, but in the setting of local inflammation, this increases to greater than 90% [29]. We demonstrate that irradiation of mice leads to a long lasting inflammatory state in the skin and a reduction in the number of regulatory T cells, which is greater than the loss of other types of CD4^+^ T cells. In the pig model, we used electron radiation that models the distribution and relative energy spectrum of SPE protons. This results in the deposition of the majority of prescribed radiation, and subsequent direct effects, primarily in the skin. An increase in immune reactivity in pig skin, as measured by response to PHA and LPS (Figure 2) was observed. In the mouse model using quantitative PCR analyses on skin samples, the gene expression patterns for chemokines capable of recruiting lymphocytes remained reduced until day 14. Murine intradermal PHA and DTH responses post-irradiation were measured and, similar to the observed responses in pigs, were elevated at 2, 4, and 7 days post-irradiation and were variable at day 14 (Figure 3 and Figure S1), suggesting different kinetics of skin inflammation post-irradiation in mice and pigs, although we cannot exclude differential effects due to the different dose distributions and types of radiation each received.

We observed a loss of all cell types in the spleen with B cells > T cells > NK cells after irradiation of mice, as has been observed in other studies [30]. We also observed a relative increase in regulatory T cells in the spleen. This resulted in a reduction in T cell proliferation in response to anti-CD3 (Figure 5). In the skin, a selective loss of regulatory T cells was observed with an increase in CD4^+^ T cell proliferation in response to anti-CD3 (Figure 4) and an increased DTH response (Figure 3). These data were generated using equal numbers of total cells in the well, but one could argue that as there was a significant loss of T cells per area (approximately 50%), the number of proliferating T cells per skin area is not increased post-irradiation. We would counter that while there is a reduction in the number of T cells in both skin and spleen, the increased proliferation of skin T cells is representative of and consistent with the increase in the PHA and LPS responses observed in the pigs (Figure 2) and DTH and PHA responses in mice (Figure 3). This could be envisioned by considering the effect of regulatory T cells on other cells, including dendritic cells, macrophages, NKT cells, γδ T cells, innate T cells, and mast cells in the skin [31]–[33] that have different sensitivities to irradiation.

There are few studies of the effects of ionizing radiation, either skin only or total body, on skin immune function. Studies of ultraviolet (UV) radiation (A and B wavelengths) have demonstrated both systemic immune suppression and a reduction in DTH responses (reviewed in [34], [35]). Most studies of UV radiation identify regulatory T cells as one cause for reduced immune responsiveness (reviewed in [36]). Our data similarly demonstrate an increase in regulatory T cells in systemic lymphoid organs that is associated with a decrease in T cell response to stimulation. Instead of a loss of DTH-like response, we observed an enhancement in both mice and miniature pigs and with exposure of both particle (electron and proton) and electromagnetic (gamma) radiation. The potential mechanisms for this difference are under study and likely include the depth of penetration, (UVB effects occur in the epidermis [reviewed in [37], [38]); the type of cellular and nucleic acid damage; and the interactions and signaling between damaged and surrounding cells. In a recent study, the effect of gamma radiation on skin allograft survival was analyzed. McFarland et al. [39] observed that at high doses (greater than 4.5 Gy), there was a decreased rate of rejection of skin allografts that was mediated by regulatory T cells. This increased survival of the allograft was only observed starting at 3 weeks post irradiation. They similarly found a relative increase in regulatory T cells in the spleen, but found an increased amount of FoxP3 mRNA in skin allografts. The prolonged survival was only observed if the mice had been previously primed with splenocytes from the strain donating the allograft. We do not believe these data contradict our data, as their finding required at least 3 weeks after irradiation to be observed and prior sensitization with donor splenocytes. In addition, an allograft rejection that likely requires memory regulatory T cells is substantially different from a DTH response. Our study is in agreement with studies that show a relative increase in splenic regulatory T cells after irradiation [40]–[45].

The radiation oncology literature contains numerous examples of local radiation leading to regression of metastatic disease present in non-irradiated regions, known as an abscopal effect [3], [8], [46]–[51]. The mechanisms for this include the potential that local radiation leads to both a loss of tumor induced regulatory T cells and an adjuvant effect in the tumor due to cell necrosis, which leads to an enhanced tumor-specific immune response. Our data demonstrate a selective loss of regulatory T cells in the skin after a 2.0–25 Gy dose of radiation has been delivered, with an enhanced local T cell response, which supports this mechanism for the abscopal effect.

We do not know if the loss of Tregs in the skin and gain in the spleen is dependent. We believe this increase was due to trafficking from the inflamed skin. Studies are mixed on the relative radiosensitivity of Tregs ([41]–[45], [52]–[56]. Some have identified increases in Treg content in mouse spleen after radiation due to reduced radiation sensitivity of Tregs [42], [44], although this was determined in vitro, where the Tregs were deprived of IL-2, which is critical in maintaining their high level of proliferation. Regulatory T cells from different tissues have different properties, cytokine dependencies, and become specialized for diverse environmental settings, tailoring their homeostatic properties and functions to a range of tissues and conditions (reviewed in [57], [58]). For an astronaut exposed to SPE radiation, enhanced skin reactivity in the setting of suppressed systemic immunity would be observed. This could result in reduced pathogen entrance through the skin, but may also result in a breakdown in the integrity of the skin, as we observed ulceration only after irradiation. The potential transient reduction in systemic immunity, while not severe, could affect immune control at all sites below the skin. Limited experiments with SOBP proton radiation to mice demonstrated a similar enhancement in DTH response in skin.

The skin is a complex organ that performs many different functions that are critical to life. The consequences of dysregulation of immune function and its effect on other functions can be disastrous, especially for an astronaut with no access to advanced medical care. Our data demonstrate that gamma rays, protons, and electrons at doses commonly used therapeutically, result in a selective depletion of regulatory T cells from the skin. Our data also identify a role for radiation-induced alterations in skin regulatory T cells and its effect on immune homeostasis. Further studies are needed to determine the mechanisms for the selective loss and the potential significance to astronauts exposed to SPE radiation, as well as, patients treated with radiation as part of cancer therapy or interventional radiology procedures [3].

## Supporting Information

Figure S1 **DTH and intradermal PHA responses in mice are enhanced after exposure to radiation.** ICR mice were stimulated with ovalbumin in CFA, rested for 21 days, irradiated with 2 Gy of proton radiation and injected in the ears with PBS or ovalbumin at the indicated days post-irradiation (A) or injected intradermally in the skin of the back with PHA or PBS (B). Ear and skin swelling was measured with an electronic digital micrometer and calculated, as described [18]. Five animals per time point were averaged and error bars represent the SEM. Statistical significance compared to pretreatment values was determined by Student’s t-test, * = p<0.05.
(TIF)
