# Supplementary figures and images for: Ionizing Radiation Selectively Reduces Skin Regulatory T Cells and Alters Immune Function
Source: PLoS One. 2014 Jun 24;9(6):e100800. doi: 10.1371/journal.pone.0100800 (PMC4069168; doi:10.1371/journal.pone.0100800)

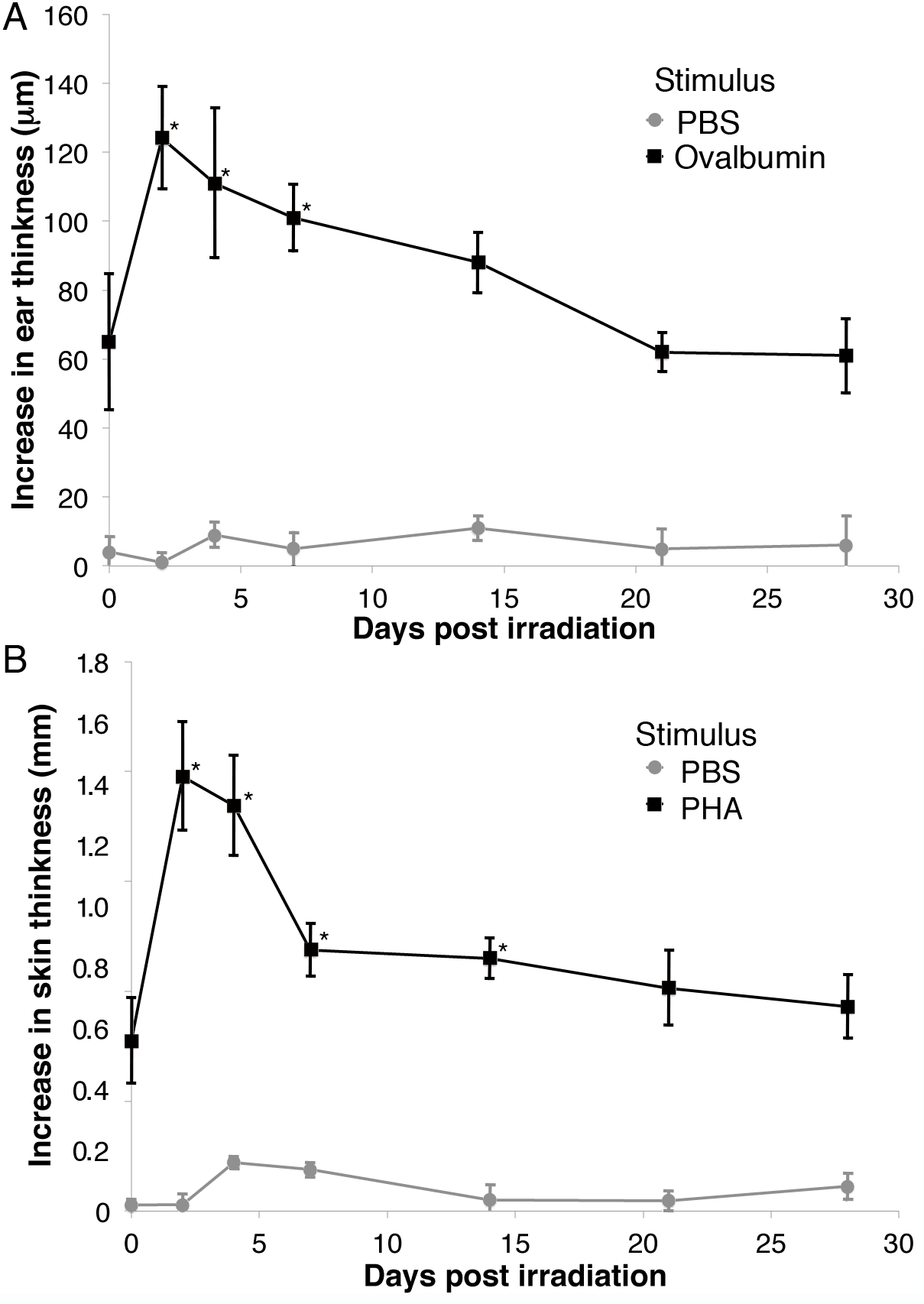

Supplement: Figure S1 — DTH and intradermal PHA responses in mice are enhanced after exposure to radiation. ICR mice were stimulated with ovalbumin in CFA, rested for 21 days, irradiated with 2 Gy of proton radiation and injected in the ears with PBS or ovalbumin at the indicated days post-irradiation (A) or injected intradermally in the skin of the back with PHA or PBS (B). Ear and skin swelling was measured with an electronic digital micrometer and calculated, as described [18]. Five animals per time point were averaged and error bars represent the SEM. Statistical significance compared to pretreatment values was determined by Student’s t-test, * = p<0.05. (TIF) [file pone.0100800.s001.tif]
